# Fast motion-compensated reconstruction for 4D-CBCT using deep learning-based groupwise registration

**DOI:** 10.1088/2057-1976/ad97c1

**Published:** 2024-12-23

**Authors:** Zhehao Zhang, Yao Hao, Xiyao Jin, Deshan Yang, Ulugbek S Kamilov, Geoffrey D Hugo

**Affiliations:** 1 Department of Radiation Oncology, Washington University School of Medicine in St. Louis, St. Louis, MO, United States of America; 2 Department of Radiation Oncology, Duke University School of Medicine, Durham, NC, United States of America; 3 Department of Electrical and Systems Engineering, Washington University in St. Louis, St. Louis, MO, United States of America; 4 Department of Computer Science and Engineering, Washington University in St. Louis, St. Louis, MO, United States of America

**Keywords:** 4D-CBCT, motion compensation, deep learning, image registration

## Abstract

*Objective*. Previous work has that deep learning (DL)-enhanced 4D cone beam computed tomography (4D-CBCT) images improve motion modeling and subsequent motion-compensated (MoCo) reconstruction for 4D-CBCT. However, building the motion model at treatment time via conventional deformable image registration (DIR) methods is not temporally feasible. This work aims to improve the efficiency of 4D-CBCT MoCo reconstruction using DL-based registration for the rapid generation of a motion model prior to treatment. *Approach.* An artifact-reduction DL model was first used to improve the initial 4D-CBCT reconstruction by reducing streaking artifacts. Based on the artifact-reduced phase images, a groupwise DIR employing DL was used to estimate the inter-phase motion model. Two DL DIR models using different learning strategies were employed: (1) a patient-specific one-shot DIR model which was trained from scratch only using the images to be registered, and (2) a population DIR model which was pre-trained using collected 4D-CT images from 35 patients. The registration accuracy of two DL DIR models was assessed and compared to a conventional groupwise DIR approach implemented in the Elastix toolbox using the publicly available DIR-Lab dataset, a Monte Carlo simulation dataset from the SPARE challenge, and two clinical cases. *Main results.* The patient-specific DIR model and the population DIR model demonstrated registration accuracy comparable to the conventional state-of-the-art methods on the DIR-Lab dataset. No significant difference in image quality was observed between the final MoCo reconstructions using the patient-specific model and population model for motion modeling, compared to using the conventional approach. The average runtime (hh:mm:ss) of the entire MoCo reconstruction on SPARE dataset was reduced from 01:37:26 using conventional DIR method to 00:10:59 using patient-specific model and 00:01:05 using the pre-trained population model. *Significance.* DL-based registration methods can improve the efficiency in generating motion models for 4D-CBCT without compromising the performance of final MoCo reconstruction.

## 1. Introduction

To resolve tissue motion for image guidance, four dimensional cone beam computed tomography (4D-CBCT) has been developed to provide a series of time-resolved volumetric images (Sonke *et al* 2005). In 4D-CBCT, acquired cone-beam projections are first sorted into different phase bins. Then multiple phase-correlated images can be reconstructed from corresponding projections to represent different respiratory phases. For a typical clinical CBCT scan, the phase binning process will lead to a large angular spacing between projections, and thus result in images severely affected by streaking artifacts when the clinically standard Feldkamp–Davis–Kress (FDK) reconstruction method (Feldkamp *et al* 1984) is used.

Without significantly extending the scan time, various methods have been proposed to improve the image quality of 4D-CBCT. One category of these methods employs iterative techniques while imposing additional constraints on the underlying results based on prior knowledge. For example, Leng *et al* (2008) proposed the prior image constraint compressive sensing (PICCS) by adding penalties on the total variation of each phase image and its difference from a prior image reconstructed using all projections. Mory *et al* (2014) reconstructed all phase images at once using spatial and temporal regularization (ROOSTER). However, these iterative reconstructions can be plagued by the residual motion and the loss of contrast for fine structures due to over-smoothing (Bergner *et al* 2010).

As an alternative strategy, motion-compensated (MoCo) reconstruction leverages a motion model generated as inter-phase deformation vector fields (DVFs) to compensate for the respiratory motion during reconstruction (Rit *et al* 2009, Brehm *et al* 2013, Wang and Gu 2013). To minimize the impact of motion pattern changes between planning and treatment delivery, motion models can be estimated from on-board 4D-CBCT images using deformable image registration (DIR). However, this data-driven method can be hampered by inaccurately estimated motion models resulting from the low quality of initially reconstructed 4D-CBCT images.

Recently, various deep learning (DL) methods have been investigated to enhance 4D-CBCT reconstruction. Most of these studies used DL models as post-processing, applying image processing to initially reconstructed images to obtain enhanced image quality. For example, Dong *et al* (2022) proposed an unsupervised DL framework to achieve 4D-CBCT artifact reduction. Zhang *et al* (2022) trained a patient-specific model using intra-patient data to enhance 4D-CBCT. Jiang *et al* (2022) proposed a feature-compensated deformable convolutional network (FeaCo-DCN) to perform interphase motion compensation in the latent feature space and generate high-quality 4D-CBCT images. However, these reconstruction methods did not incorporate the physical modeling of CBCT imaging and were performed entirely in the image domain based on the input images, making it difficult to restore information that was already lost in the poor-quality initial images. We previously proposed a DL-assisted motion compensation method to enhance the reconstruction quality by providing artifact-reduced initial images for motion modeling (Zhang *et al* 2023). Specifically, a pre-trained convolutional neural network (CNN) was used to remove the streaking artifacts in FDK-reconstructed initial images. Based on the artifact-reduced images, a conventional registration algorithm was then employed to build the motion model. The final 4D-CBCT images were generated using MoCo reconstruction with the improved motion model. Results have demonstrated that conducting MoCo reconstruction with CNN-generated high-quality initial images is more advantageous than directly using initial images for MoCo or utilizing the network solely as a post-processing operator. However, the clinical practice of MoCo-based reconstruction is still limited by the long computation time of conventional DIR algorithms, which estimate the optimal transformation for each pair of images by solving an iterative optimization problem (Sotiras *et al* 2013). For example, a pairwise registration running on CPU can take minutes to hours to finish (Klein *et al* 2009). Due to the intrinsic temporal coherence in 4D-CBCT images, groupwise registration has shown additional benefits by employing a joint optimization to register all images simultaneously (Metz *et al* 2011). Nevertheless, this comes at the expense of even higher computational demands. With conventional DIR approaches, building a motion model after 4D-CBCT acquisition takes minutes to hours, clearly infeasible with image-guided treatment where the MoCo image is required immediately after 4D-CBCT acquisition.

DL-based registration methods have also gained increasing popularity, especially for unsupervised approaches that do not require ground-truth deformation fields for training. Balakrishnan *et al* (2019) proposed VoxelMorph based on a U-Net like architecture and spatial transform network (Jaderberg *et al* 2015) to implement fast pairwise registration and achieved comparable performance to the traditional registration methods. In the field of groupwise registration, Che *et al* (2019) proposed a deep groupwise registration network with the principal component analysis-generated template for fundus images. Zhang *et al* (2021) proposed GroupRegNet to achieve groupwise 4D image registration using a one-shot learning strategy, which eliminated the requirement for abundant training data. However, GroupRegNet did not take full advantage of DL’s low inference time because the model needed be trained from scratch for each set of new images.

The goal of this study is to produce a nearly real-time MoCo process capable of building a motion model and reconstructing a MoCo 4D-CBCT in seconds. Specifically, instead of using a conventional iterative registration process to build a motion model, we propose to use DL-based groupwise registration for motion modeling based on artifact-reduced initial images to achieve a fast MoCo reconstruction for 4D-CBCT. We summarize the major contributions of this study in three aspects. Firstly, to the best of our knowledge, this study is the first to integrate DL-based registration methods into the MoCo framework for achieving fast 4D-CBCT reconstruction. The performance was evaluated using both simulated data and real patient data. The comparison with other reconstruction methods was also performed. Secondly, both a patient-specific model and a population-based model were employed for the registration purpose. The patient-specific model does not require a large dataset for training, while the population model benefits from the low inference time of DL. The registration accuracy and subsequent reconstruction performance of these two models was compared. Thirdly, the proposed method enables a fast and high-quality MoCo reconstruction for 4D-CBCT imaging using a clinical standard 1-min scan, making it suitable for clinical applications.

## 2. Methods

### 2.1. Overview

**Figure 1.**
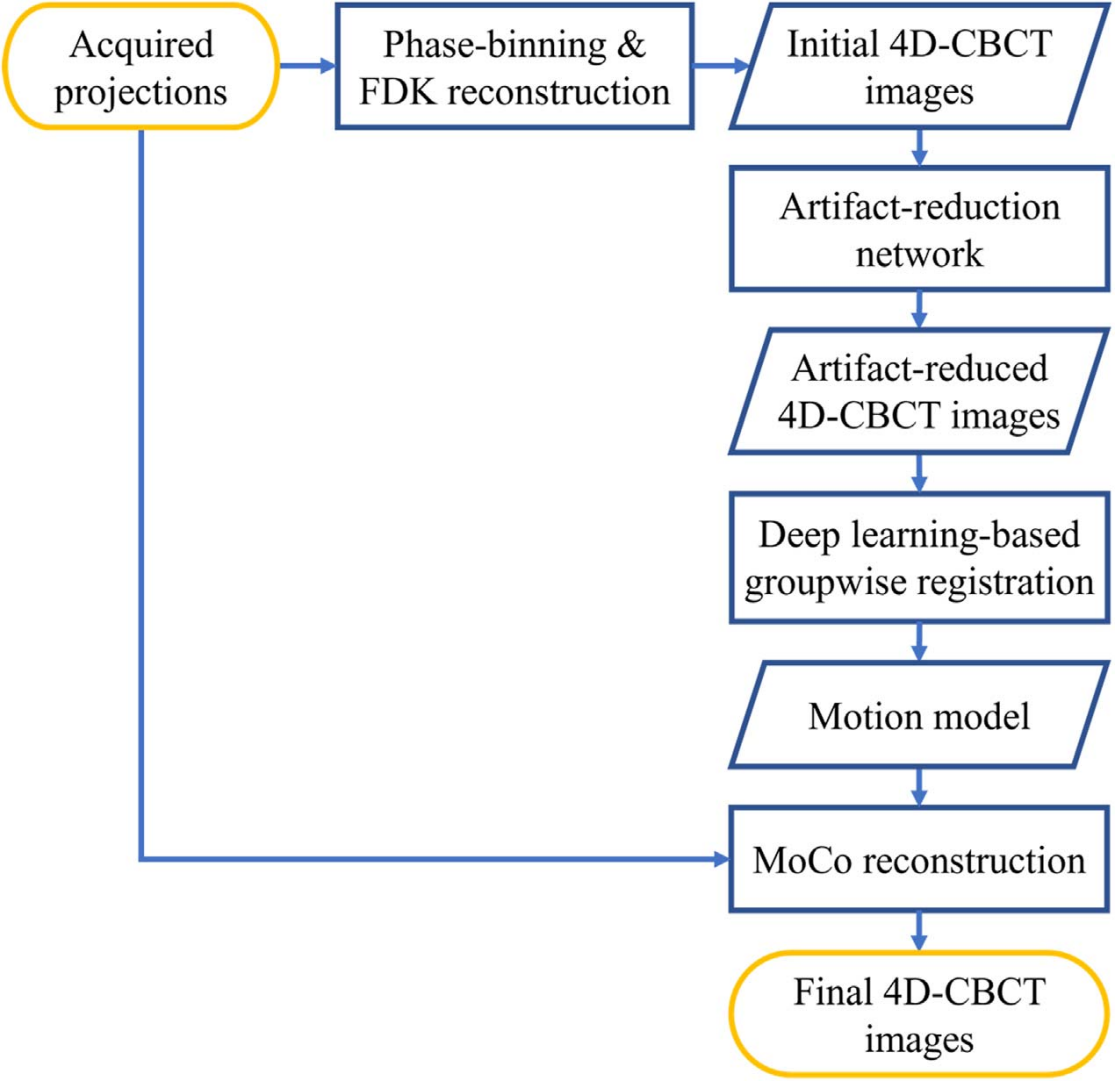
Flowchart of the proposed fast 4D-CBCT reconstruction using DL-based artifact reduction and groupwise registration.

Our proposed method combined an artifact-reduction network and a groupwise registration network to achieve fast 4D-CBCT reconstruction. The overall workflow is shown in figure 1. The initial 4D-CBCT images were first reconstructed using phase-binned projections and the FDK algorithm. Then, as in our previous work, a pre-trained artifact-reduction DL model was employed to improve the image quality to a level useful for motion model generation. DVFs between the artifact-reduced 4D-CBCT images were efficiently computed using a DL-based groupwise registration model, representing the motion model between different respiratory phases. Subsequently, MoCo reconstruction was performed for each phase by deforming other phases into the target phase using warped backprojection trajectories based on the motion model, as originally described by Rit *et al* (2009). Utilizing the accurately estimated motion model and MoCo reconstruction, all projection information was combined to solve the undersampling problem for 4D-CBCT resulting from phase binning. Our source code and model parameters are available at https://github.com/zhangzhehao95/Fast_4DCBCT_DLreg.

Two separate groupwise registration models were trained and evaluated, a patient-specific model and a population model. The patient-specific model was trained using patient-specific artifact-reduced 4D-CBCT images. The population model was trained beforehand using multiple sets of pre-collected 4D images. The population model needs only to be applied to the new patient’s 4D-CBCT images to infer a DVF, which is substantially faster than training or updating a patient-specific model on the fly. However, the population model may not be as accurate on a patient-by-patient basis compared to the patient-specific model.

### 2.2. Datasets and pre-processing

A free-breathing CBCT dataset and a 4D-CT dataset were collected under Institutional Review Board approval, in accordance with the Declaration of Helsinki. The CBCT dataset consisted of 18 patients undergoing radiotherapy for lung and upper abdomen tumors in our institution. Three CBCT scans from each patient’s treatment session were included. The projection images were obtained on a Varian Edge system (Varian Medical Systems, Palo Alto, US) using a standard 60 s CBCT scan protocol. Each scan acquired approximately 900 projections with a matrix size of 1024 × 768 and a pixel spacing of 0.338 × 0.338 mm^2^ using a single 360° gantry rotation and half-fan mode. The source-to-isocenter distance (SID) and source-to-detector distance (SDD) were 1000 mm and 1500 mm. The acquired projections were sorted into 10 phases for each scan based on the respiratory signal, which was extracted using the Amsterdam Shroud method (Zijp *et al* 2004) with the Reconstruction Toolkit (RTK) implementation (Rit *et al* 2014). 16 patients were randomly selected for the artifact-reduction network training and validation, and the two remaining patients were used for the evaluation of the proposed MoCo reconstruction pipeline. By default, all CBCT images were reconstructed to 224 × 224 × 96 voxels with a spacing of 2 × 2 × 2 mm^3^.

The 4D-CT dataset was collected from 35 patients, acquired on a SOMATOM Confidence scanner (Siemens, Germany). The initially constructed in-plane image size was 512 × 512 with a pixel spacing ranging from 0.98 × 0.98 to 1.56 × 1.56 mm^2^ for different patients. The number of slices varied from 133 to 270 with a fixed thickness of 2 mm. The 4D-CT images were resampled and cropped to maintain the same size and resolution of above-mentioned CBCT images. The image intensity values were normalized to the range of [0, 1] after clipping to the 0.5 and 99.5 percentiles of the intensity distribution. After pre-processing, this dataset was used to train our population registration model.

To conduct hyperparameter selection and evaluation for the registration models, the publicly available DIR-Lab dataset (Castillo *et al* 2009) was used. The DIR-Lab dataset consists of 10 thorax 4D-CT scans, each with 300 pairs of landmarks manually delineated at both end-inhalation and end-exhalation phases. The registration accuracy was assessed in terms of target registration error (TRE) calculated using the corresponding landmarks. The first three cases of DIR-Lab datasets were used for validation, while the remaining seven cases (4–10) were used for testing.

In addition to the clinical free-breathing CBCT dataset, a Monte Carlo simulation dataset of 9 patients from the public sparse-view reconstruction (SPARE) challenge (Shieh *et al* 2019) was also used for the evaluation of reconstruction performance. For the simulation dataset, the cone-beam projections were simulated with scatter and quantum noise using ground truth 4D-CT images. Each dataset consisted of 680 evenly distributed projections over 360° rotation, coupled to a 1-min long real-time position management (RPM) trace. The matrix size and pixel spacing of simulated projections were 512 × 384 and 0.776 × 0.776 mm^2^, respectively. Different region-of-interest (ROI) masks of body, lung, planning target volume (PTV) and bony anatomy were provided for each patient. Using the ground truth 4D-CTs, quantitative analysis is available for the simulation dataset.

### 2.3. Artifact-reduction network

To ensure the accuracy of data-driven motion modeling and the performance of MoCo reconstruction, high-quality initial 4D-CBCT images are necessary. As proposed in our previous work (Zhang *et al* 2023), an artifact-reduction network was trained to learn the relationship between streaking artifacts-corrupted CBCT images and corresponding high-quality images with the same anatomical information and respiratory state. Because of the lack of high-quality 4D-CBCT images in the clinical setting, we trained the artifact-reduction network in a self-contained manner (Madesta *et al* 2020) by constructing additional pseudo-average CBCT images. Unlike the phase binning method utilized in normal 4D-CBCT reconstruction, each pseudo-average CBCT phase bin consisted of projections from different respiratory phases across different respiratory cycles. Correspondingly, the reconstructed pseudo-average CBCT images had the same averaged respiratory motion as the time-average 3D-CBCT image since projections from all different respiratory phases were used in the reconstruction. Meanwhile, the pseudo-average CBCTs also exhibited equivalent artifact pattern and image quality as the initial 4D-CBCT images due to their similar undersampled projection distributions. Based on this characteristic, the network was trained to reduce streaking artifacts by using the time-average CBCT and pseudo-average CBCT as training pair. The well-trained network was used to generate artifact-reduced initial 4D-CBCT images by processing the FDK-reconstructed initial 4D-CBCT images as input. More details of the training configurations can be found in Zhang *et al* (2023). The CNN-generated artifact-reduced initial images were used for the subsequent motion modeling.

### 2.4. Motion modeling using deep learning-based groupwise registration

Let ${{I}}^{{N}}{=}\left\{{{I}}_{{n}}{\in }{{{\mathbb{R}}}}^{{3}},|,{n}{=}{1}{,}{\ldots }{,}\,{N}\right\}$ represents a set of artifact-reduced initial 4D-CBCT phase images. In this study, we focused on the case ${N}{=}{10}$ which means that the respiratory cycle was divided into 10 phases. The motion modeling process aims to estimate a transformation ${{T}}_{{i}}^{{j}}$ that maps the coordinate frames of each pair of phase images ${{I}}_{{i}}$ and ${{I}}_{{j}}$ so that

\begin{eqnarray*}{I}_{i}={I}_{j}^\circ {T}_{i}^{j},\end{eqnarray*}

where ${I}_{j}\circ {T}_{i}^{j}$ represents ${I}_{j}$ warped by ${T}_{i}^{j}$.

To enforce the temporal coherence in deformations across the 4D-CBCT sequence and to avoid the bias introduced by selecting a specific phase image as the reference, we employed groupwise registration with an implicit template (Metz *et al* 2011). Specifically, a set of transformations ${{T}}_{{{tem}}}^{{N}}{=}\left\{{{T}}_{{{tem}}}^{{n}}{\in }{{{\mathbb{R}}}}^{{3}}{\times }{{{\mathbb{R}}}}^{{3}},|,{n}{=}{1}{,}{\ldots }{,}\,{N}\right\}$ were estimated to warp each phase image ${{I}}_{{n}}$ to the common template ${{I}}_{{{tem}}}$ by solving:

\begin{eqnarray*}\mathop{{argmin}}\limits_{{T}_{{tem}}^{N}}\,L\left({I}^{N},{T}_{{tem}}^{N}\right)\,\end{eqnarray*}

\begin{eqnarray*}=\,\mathop{{argmin}}\limits_{{T}_{{tem}}^{N}}\,{L}_{{sim}}\left({I}^{N}\circ {T}_{{tem}}^{N},{I}_{{tem}}\right)+{L}_{{smo}}\left({T}_{{tem}}^{N}\right)\end{eqnarray*}

\begin{eqnarray*}+{L}_{{con}}\left({T}_{{tem}}^{N}\right)\,\end{eqnarray*}

where ${I}^{N}\circ {T}_{{tem}}^{N}$ represents warped input images and ${I}_{{tem}}=\frac{1}{N}{\sum }_{n}({I}^{n}\circ {T}_{{tem}}^{n})$ is the common implicit template by averaging all warped input images. ${L}_{{sim}}$ represents the similarity loss that measures difference in appearance between each warped phase image and the template, ${L}_{{smo}}$ denotes the smoothness regularization that penalizes sharp variations in predicted transformations, and ${L}_{{con}}$ is the zero average deformation constraint that ensures the implicit template lies at the center of the inputs.

**Figure 2.**
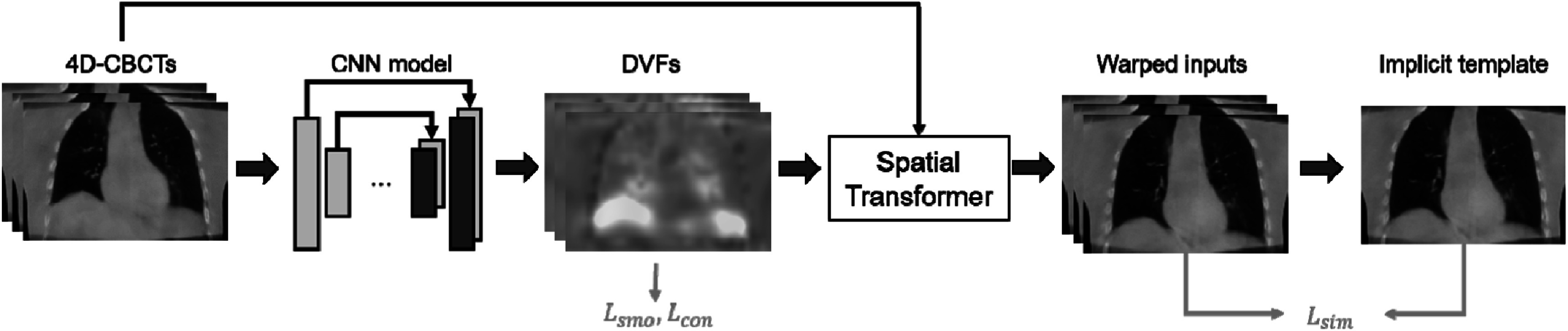
Framework of the DL-based groupwise registration.

Inspired by Zhang *et al* (2021), DL was employed to solve the groupwise registration. As shown in figure 2, a CNN was used to generate a set of DVFs, which were then applied to the input 4D-CBCT images through the spatial transformer network. The CNN-generated DVFs were expected to warp all inputs to the same appearance, as the similarity loss was minimized when each single warped input matched the averaged implicit template. It should be noted that the direct output of the network is the DVF between inputs and the implicate temple ${{D}}_{{{tem}}}^{{N}}$ rather than the transformation ${{T}}_{{{tem}}}^{{N}}$, which can be related through ${{T}}_{{{tem}}}^{{N}}\left({{\bf{p}}}\right){=}\,{{D}}_{{{tem}}}^{{N}}\left({{\bf{p}}}\right){+}{{\bf{p}}}$ for each voxel **p**. To generate the motion model for MoCo reconstruction, inter-phase transformation fields between arbitrary phase ${i}$ and phase ${j}$ were obtained by compositing ${{T}}_{{{tem}}}^{{j}}$ and the inverse transformation (Chen *et al* 2008) of ${{T}}_{{{tem}}}^{{i}}$:

\begin{eqnarray*}{T}_{i}^{j}\left(p\right)={T}_{tem}^{j}\left({T}_{i}^{tem}\left(p\right)\right)={T}_{tem}^{j}\left({T}_{tem}^{i-1}\left(p\right)\right).\end{eqnarray*}

#### 2.4.1. Network architecture

**Figure 3.**
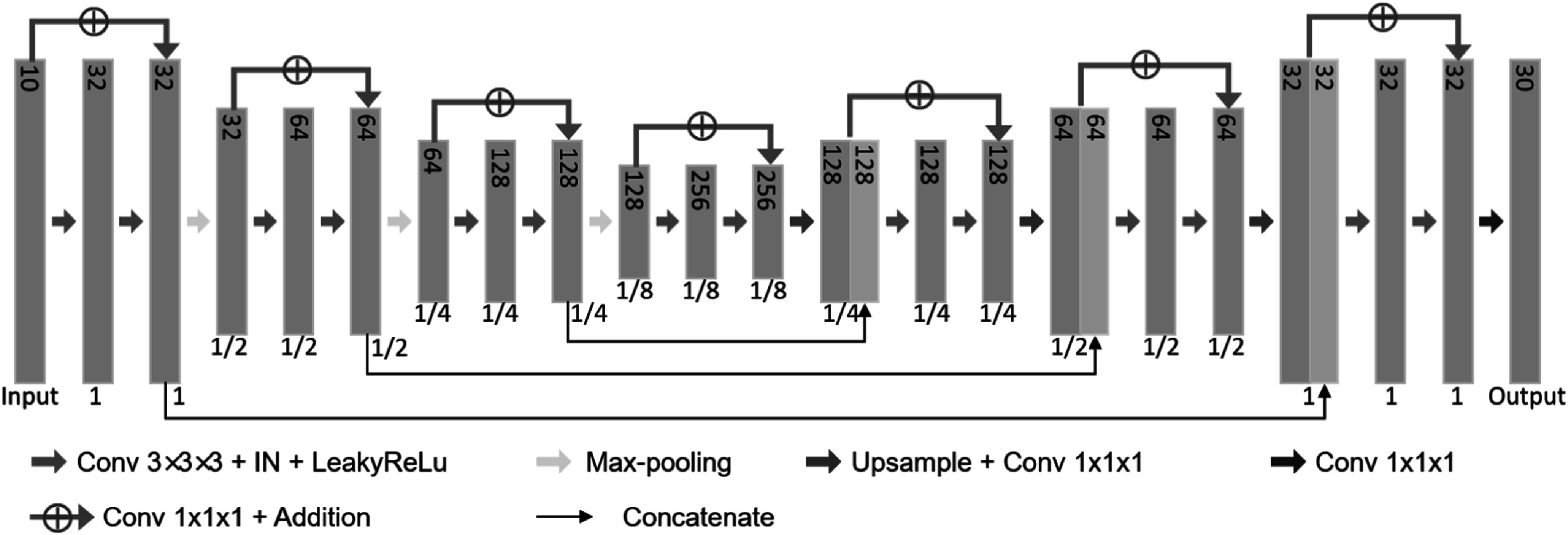
Architecture of the CNN model used for deformation vector fields generation. The number of channels is indicated on the upper side of each feature map, and the spatial resolution with respect to the input is denoted underneath.

The utilized CNN model was based on the design of GroupRegNet (Zhang *et al* 2021), with minor modifications made according to validation performance. The detailed architecture of the CNN model is shown in figure 3, which was a combination of 3D U-Net (Çiçek *et al* 2016) and ResNet (He *et al* 2016). The input was formed by concatenating all 10 phase images into a 4D volume along the channel dimension. Under the U-Net framework, four residual blocks were used for the encoding stage and three residual blocks were used for the decoding stage. 3D convolution with a kernel size of 3 was used for each layer in the residual block, followed by an instance normalization (IN) and a leaky version of ReLU (LeakyReLU) with parameter 0.2. The output channel number was mapped to 30 to represent the displacement along three spatial directions for each of the 10 input images.

#### 2.4.2. Loss function

The overall loss function for the model training followed the optimization problem of groupwise registration as presented in equation (2), which consisted of the similarity loss ${{L}}_{{{sim}}}$, smoothness regularization ${{L}}_{{{smo}}}$ and zero average deformation constraint ${{L}}_{{{con}}}$. The similarity loss was measured using the local normalized cross-correlation (LNCC) coefficient:

\begin{eqnarray*}\begin{array}{c}{LNCC}\left(f,g\right)\\ =\,\displaystyle \sum _{{\bf{p}}\in {\mathrm{\Omega }}}\frac{{\left({\sum }_{{{\bf{p}}}_{i}}(f({{\bf{p}}}_{i})-\mathop{f}\limits^{\unicode{773}}({\bf{p}}))(g({{\bf{p}}}_{i})-\mathop{g}\limits^{\unicode{773}}({\bf{p}}))\right)}^{2}}{({\sum }_{{{\bf{p}}}_{i}}{(f({{\bf{p}}}_{i})-\mathop{f}\limits^{\unicode{773}}({\bf{p}}))}^{2})({\sum }_{{{\bf{p}}}_{i}}{(g({{\bf{p}}}_{i})-\mathop{g}\limits^{\unicode{773}}({\bf{p}}))}^{2})},\end{array}\end{eqnarray*}

where $\mathop{f}\limits^{\unicode{773}}\left({\bf{p}}\right)$ and $\mathop{g}\limits^{\unicode{773}}({\bf{p}})$ denotes the mean voxel value within a local window of size ${n}^{3}$ centered at voxel ${\bf{p}}$. $n=5$ was used in this study. The total similarity loss was determined by computing the average negative LNCC coefficients across each warped input image and the implicit template, as a lower LNCC value indicates higher similarity:

\begin{eqnarray*}\begin{array}{c}{L}_{{sim}}\left({I}^{N}\circ {T}_{{tem}}^{N},{I}_{{tem}}\right)\\ =\,-\frac{1}{N}\displaystyle \sum _{n}{LNCC}\left({I}^{n}\circ {T}_{{tem}}^{n},\,{I}_{{tem}}\right).\end{array}\end{eqnarray*}

Since smooth deformation fields are expected in both spatial and temporal dimensions, a bending energy regularizer on spatial gradients and a diffusion regularizer on temporal gradients were used to constrain the smoothness:

\begin{eqnarray*}\begin{array}{ccl}{L}_{{smo}}\left({T}_{{tem}}^{N}\right) &amp; = &amp; {\lambda }_{1}{L}_{{spatial}}\left({T}_{{tem}}^{N}\right)\\ &amp; &amp; +\,{\lambda }_{2}{L}_{{temporal}}\left({T}_{{tem}}^{N}\right)\\ &amp; = &amp; {\lambda }_{1}\frac{1}{3N\left|{\mathrm{\Omega }}\right|}\displaystyle \sum _{{\boldsymbol{n}}{\boldsymbol{,}}{\bf{p}}\in {\mathrm{\Omega }}}{\unicode{x02016}{{\mathrm{\nabla }}}^{2}{D}_{{tem}}^{n}\left({\bf{p}}\right)\unicode{x02016}}^{2}\\ &amp; &amp; +{\lambda }_{2}\frac{1}{3N{\mathrm{|}}{\mathrm{\Omega }}{\mathrm{|}}}\displaystyle \sum _{{\bf{p}}\in {\mathrm{\Omega }}}{\unicode{x02016}{{\mathrm{\nabla }}}_{n}{D}_{{tem}}^{N}\left({\bf{p}}\right)\unicode{x02016}}^{2},\end{array}\end{eqnarray*}

where ${\lambda }_{1}$ and ${\lambda }_{2}$ are regularization weights for spatial smoothness and temporal smoothness, respectively. All the gradients were approximated using forward differences.

By forcing the sum of all deformations is equal to zero, the zero average deformation constraint was introduced to implicitly define the template coordinate frame as being located at the center of all input images:

\begin{eqnarray*}{L}_{{con}}\left({T}_{{tem}}^{N}\right)={\lambda }_{3}\sqrt{\frac{1}{3{\mathrm{|}}{\mathrm{\Omega }}{\mathrm{|}}}\displaystyle \sum _{{\bf{p}}\in {\mathrm{\Omega }}}{\left(\displaystyle \sum _{n}{D}_{{tem}}^{n}\left({\bf{p}}\right)\right)}^{2}}\end{eqnarray*}

#### 2.4.3. Patient-specific model and population model

**Figure 4.**
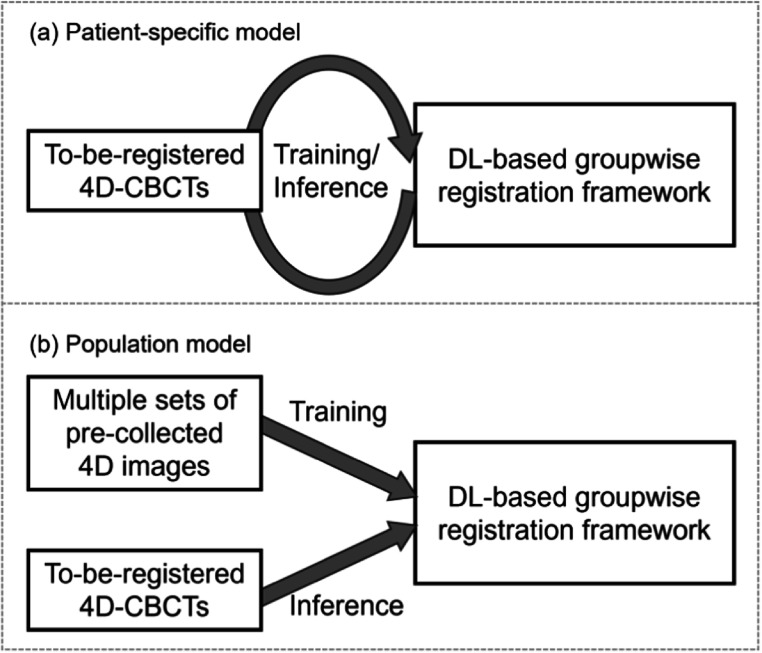
Different learning strategies for (a) the patient-specific model and (b) the population model.

In this study, both the patient-specific groupwise registration model and the population groupwise registration model were trained to estimate the motion model. These two models utilized the same registration framework as shown in figure 2 but employed different learning strategies. As depicted in figure 4(a), the patient-specific model was trained using one-shot learning. This strategy is analogous to traditional iterative optimization methods, where it seeks to derive a DVF solely from the input 4D images but replaces the optimization process with the updating of network parameters by reusing the same set of 4D images as input. The to-be-registered artifact-reduced 4D-CBCT images from a specific patient was repeatedly fed into the model. This process resulted in obtaining the corresponding motion model along with the well-trained network parameters.

Although the one-shot learning strategy can eliminate the need for abundant training data by training a patient-specific model, the parameters need to be learned from scratch when inferring on new to-be-registered images, which leads to a prolonged registration time. On the other hand, as shown in figure 4(b), the population model was pre-trained on a comprehensive dataset and can take a new set of to-be-registered images as input during the inference stage to generate DVFs in a single pass. This capability could further accelerate the motion modeling for MoCo reconstruction. Due to the difficulty of obtaining a large number of high-quality 4D-CBCT images, the population model was trained using the collected 4D-CT dataset as described above and applied to unseen artifact-reduced 4D-CBCT images for motion modeling, under the assumption that these images have a comparable image quality to that of 4D-CTs. Previous work by Teng *et al* (2021) has also demonstrated the feasibility of introducing cross-modality differences into the training and predicting stages of a registration network.

#### 2.4.4. Implementation details

Both the patient-specific model and the population model were implemented in PyTorch and trained using a NVIDIA Tesla A100 GPU. For the patient-specific model, the Adam optimizer with a learning rate of 1 × 10^−3^ was used for optimization. The batch size was set to 1. Regularization weights for spatial smoothness λ_1_, temporal smoothness regularization λ_2_, and zero average deformation λ_3_ were empirically set to 1, 1 × 10^−5^, and 5 × 10^−2^, respectively. We considered convergence to be achieved when the difference between the current loss function value and the moving average of the previous 20 iterations was less than 1 × 10^−5^.

To ensure a fair comparison between the patient-specific model and the population model, both the network architecture and hyperparameters were kept consistent across the two models. The only adjustment made for the population model was the use of a fixed 200 epochs for training.

### 2.5. Evaluation

The SPARE Monte Carlo simulation dataset and the testing data from our collected clinical CBCT dataset were used for the image quality evaluation of reconstructed 4D-CBCT images. Following the pipeline presented in figure 1, the conventional FDK reconstruction was first performed to generate phase-correlated images for each dataset. Then, the pre-trained artifact-reduction network was applied to improve the quality of the initial 4D-CBCT images. Our previous work has demonstrated the advantage of using DL-improved initial images in 4D-CBCT MoCo reconstruction (Zhang *et al* 2023). In this study, we compared the quality of MoCo reconstructed images using the motion models estimated from a conventional registration method, population groupwise model and patient-specific model. The nD + t registration algorithm (Metz *et al* 2011) with open-source Elastix implementation (Klein *et al* 2010) was employed as the reference conventional method, which also utilized an implicit template and imposed regularizations of deformations in both spatial and temporal directions. The runtime was recorded for each method.

To evaluate the reconstruction performance quantitatively, root mean square error (RMSE), peak signal-to-noise ratio (PSNR), and structural similarity index metric (SSIM) between the reconstructed 4D-CBCT images and ground truth 4D-CT images were calculated on the simulation dataset for different ROIs. Due to the imperfect calibration of projection intensity for the SPARE simulation dataset (Shieh *et al* 2019), we followed the evaluation process of SPARE challenge by applying optimal linear before the calculation to ensure a structure-based evaluation. For RMSE calculation, the mean value of reconstructed image was initially aligned with the ground truth within each ROI. Regarding SSIM calculation, a linear scaling yielding the highest SSIM was searched and then applied to the reconstructed image. Five previous works that completed the SPARE challenge, including the MC-FDK (Rit *et al* 2009), MA-ROOSTER (Mory *et al* 2016), PICCS-MoCo (Riblett *et al* 2018), MC-PICCS and prior deforming (Ren *et al* 2008), were included for performance comparison. These methods employed different reconstruction approaches, including iteration-based, MoCo-based, and prior knowledge-based methods. This diverse range allows for a more comprehensive comparison for our proposed methods, which fall under the MoCo-based approach. We directly obtained the reconstruction results of these methods from the SPARE challenge host to avoid the potential bias resulting from imperfect reproductions. To perform a valid comparison, all reconstruction results, ground truths and mask images were cropped and resampled to align with the volume size and resolution used for our CBCT reconstruction.

## 3. Results

### 3.1. Evaluation of deep learning-based groupwise registration

**Figure 5.**
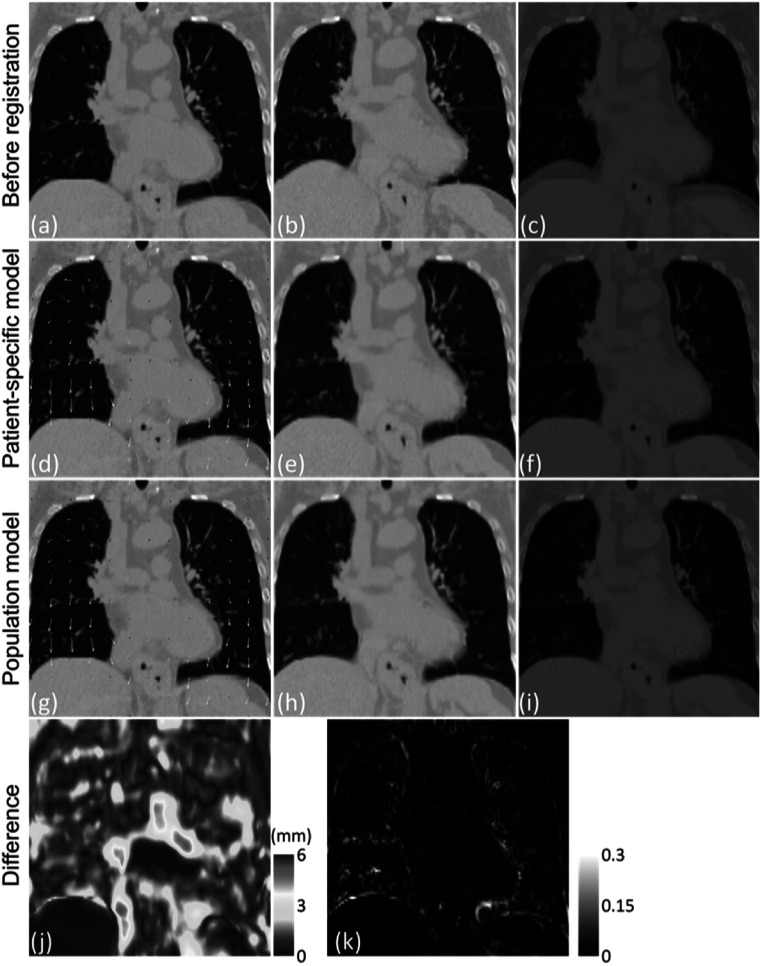
Example of the registration results of DIR-Lab case 5 using DL-based groupwise registration. The first row shows the original end-inhale phase (fixed image, a), end-exhale phase (moving image, b) and the superimposed image (c). The vector plots show the magnitude and direction of the estimated deformation fields using patient-specific model (d) and population model (g). (j) show the magnitude difference between two deformation fields. (e), (h) and (k) show the corresponding deformed images and their absolute difference. (f) and (i) show the superimposed images after registration. All CT images are normalized and displayed with a range of [0, 1].

Figure 5 presents the registration results of one example DIR-Lab case using of the patient-specific groupwise registration model and the population groupwise registration model. Although all inter-phase DVFs were simultaneously obtained by groupwise registration, the original end-inhale and end-exhale phases were considered as the fixed and moving images, respectively, for illustration. The superimposed images shows that both models can generate proper DVFs to align the moving image and fixed image. As shown in the absolute difference maps, both models generated similar registration results. The main variance in the generated DVFs and corresponding deformed images appeared at the boundary regions. Compared to the patient-specific model, slight mismatches can be observed in the results of the population model as indicated by red arrows, which is in line with our expectation that the population model may not be as accurate as the patient-specific model. This result is consistent with a previous work on a different task by Zhang *et al* (2022), which showed that the patient-specific model can outperform the population-based model in enhancing 4D-CBCT images for radiomic feature extraction.

Table 1 shows the registration accuracy on seven DIR-Lab testing cases. Although the patient-specific model obtained smaller average TRE value than the population model (Wilcoxon signed-rank, ${p}{=}{0}{.}{02}$), the average runtime of patient-specific model was 664 ± 78 iterations with approximately 2 seconds per iteration, while the population model only required one single pass. The performance of conventional Elastix registration was included as a reference. To verify the reliable registration performance of our implemented DIR models and ensure a fair evaluation, two previous learning-based registration methods, LungRegNet (Fu *et al* 2020) and GroupRegNet (Zhang *et al* 2021), were included for comparative analysis. These two methods employed the same learning strategy as the population and patient-specific models, respectively. Specifically, Elastix demonstrated an accuracy level fell between the population model and the patient-specific model. Comparing our population model to LungRegNet, both of which utilized the pre-training strategy, similar registration performance can be observed (Wilcoxon signed-rank, ${p}{=}{0}{.}{47}$). Additionally, our patient-specific model achieved comparable performance to GroupRegNet (Wilcoxon signed-rank, ${p}{=}{0}{.}{19}$), both using one-shot learning.

**Table 1.** Target registration error (mm) values for different DL-based registration methods on DIR-Lab testing cases.

DIR-Lab case	Before registration	Population model	Patient-specific model	Elastix	LungRegNet (Fu *et al* 2020)	GroupRegNet (Zhang *et al* 2021)
4	9.83 ± 4.86	1.70 ± 1.02	1.43 ± 0.96	1.48 ± 1.11	1.39 ± 0.99	1.43 ± 0.97
5	7.48 ± 5.51	1.81 ± 1.35	1.44 ± 1.22	1.67 ± 1.42	1.43 ± 1.31	1.41 ± 1.22
6	10.89 ± 6.96	1.62 ± 1.15	1.22 ± 0.63	1.27 ± 0.68	2.26 ± 2.93	1.31 ± 0.72
7	11.02 ± 7.42	1.65 ± 1.01	1.24 ± 0.67	1.31 ± 0.84	1.42 ± 1.16	1.28 ± 0.65
8	14.99 ± 9.00	2.91 ± 2.34	1.25 ± 0.72	2.71 ± 4.28	3.13 ± 3.77	1.33 ± 1.08
9	7.92 ± 3.97	1.97 ± 1.02	1.30 ± 0.74	1.27 ± 0.68	1.27 ± 0.94	1.30 ± 0.69
10	7.30 ± 6.34	1.73 ± 1.22	1.20 ± 0.68	1.25 ± 0.77	1.93 ± 3.06	1.22 ± 0.63
Mean	9.92	1.91	1.30	1.57	1.83	1.33

### 3.2. Reconstruction results of Monte Carlo simulation data

**Figure 6.**
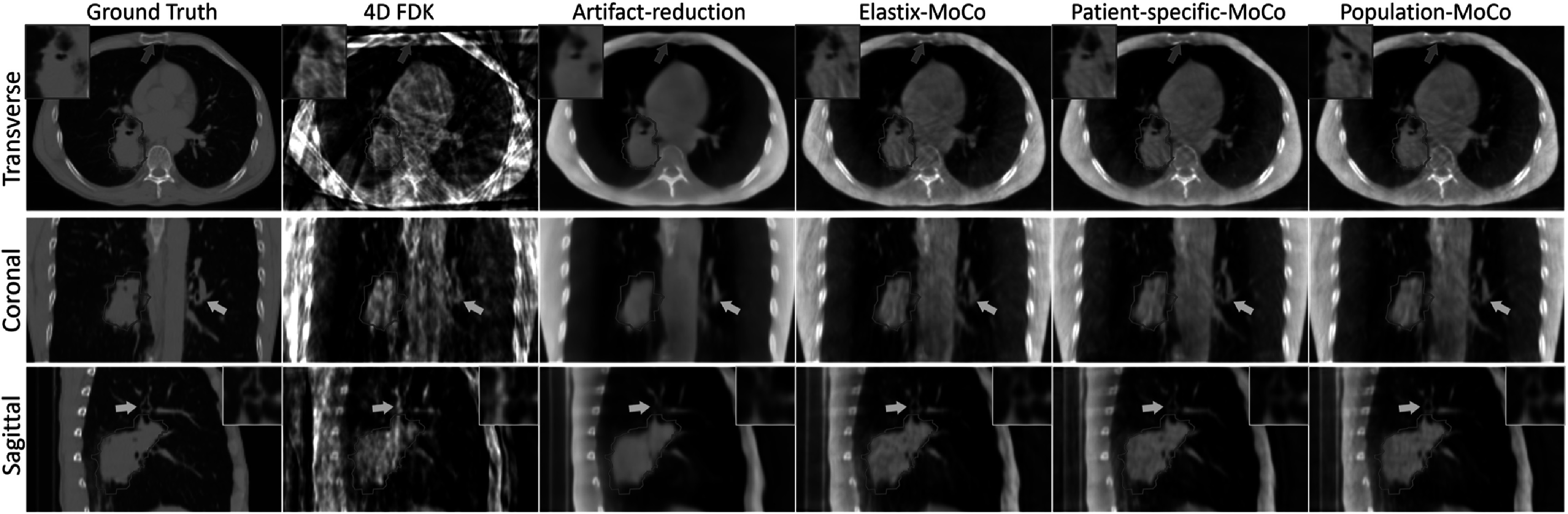
Reconstructed end-exhale phase of a SPARE Monte Carlo simulation dataset (MC_V_P2_SC_01). The planning target volumes are indicated by red contours and a close-up is provided in transverse view. The display ranges are [−0.001, 0.026] mm^−1^.

Figure 6 shows the representative reconstruction results of patient 2 from the simulation dataset. Column 1 represents the ground truth CT image used for CBCT projections simulation. Columns 2–6 show the reconstructed 4D-CBCT at end-exhale phase using FDK (4D FDK), artifact-reduction network with 4D FDK as input (artifact-reduction), and MoCo with motion models estimated from the Elastix (Elastix-MoCo), patient-specific groupwise registration model (patient-specific-MoCo) and pre-trained population groupwise registration model (population-MoCo), respectively. The image quality of 4D FDK was severely degraded due to the undersampled projections. The artifact-reduction network can remove the majority of streaking artifacts present in 4D FDK, but over-smoothness and distortion can be observed near the tumor (red contours and zoomed-in images in transverse view), bony structures (blue arrows) and vessels (yellow arrows and zoomed-in images in sagittal view). Using the artifact-reduced images for motion modeling, different registration methods achieved similar MoCo reconstruction results. Elastix-MoCo, patient-specific-MoCo and population-MoCo all restored more fine structures and further improved the image quality compared to using artifact-reduction network alone, which validated the feasibility of employing DL-based groupwise registration for MoCo reconstruction. It should be noted that brighter circular artifacts are present in all reconstruction methods. This is a known issue with the SPARE simulation dataset, potentially resulting from imperfect modeling of the half-fan bowtie filter used during synthetic projection generation.

**Figure 7.**
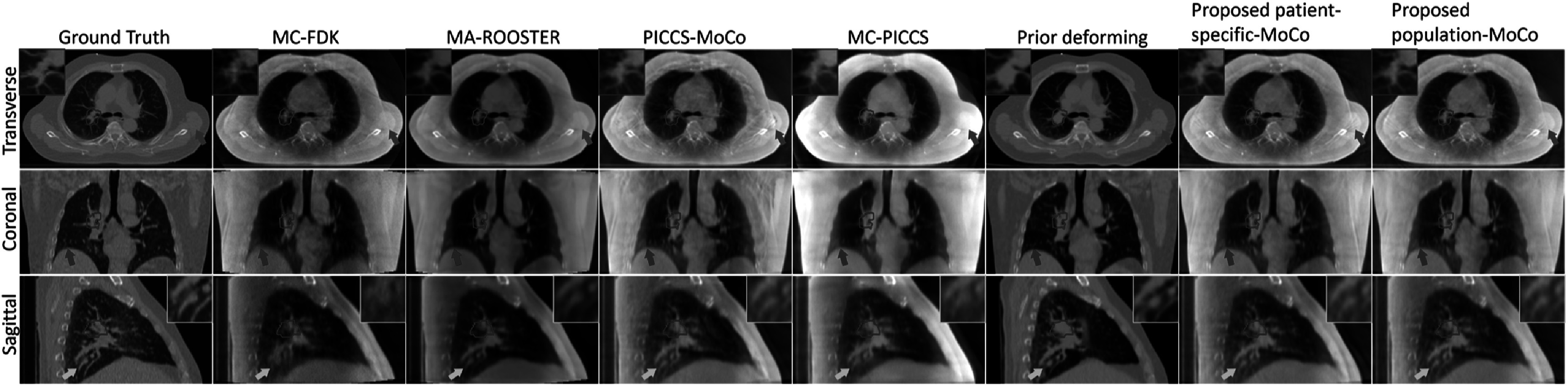
Comparison of the reconstructed end-exhale phase of simulation dataset MC_V_P4_SC_01 using five reference methods that completed the SPARE challenge as well as our proposed methods. The display ranges are [−0.001, 0.026] mm^−1^.

Figure 7 shows a representative comparison of reconstructed 4D-CBCT images using different methods. In particular, the prior deforming method achieved better consistency in image values with the ground truth, as the pretreatment 4D-CT images were deformed to generate the results. However, obvious structure variations were observed due to the changes of patient anatomy and position across different scans, as highlighted by red contours and the zoomed-in images. Noticeable blurring artifacts can be observed in MC-FDK and MC-PICCS at the diaphragm (red arrows) and vessel regions (yellow arrows and zoomed-in images in sagittal view). Additionally, MC-PICCS presented more pronounced attenuation coefficient variation compared to the ground truth. The proposed patient-specific-MoCo and population-MoCo achieved comparable image quality to PICCS-MoCo and MA-ROOSTER. It is worth noting that the PICCS-MoCo was similar to our proposed method but used PICCS algorithm for initial 4D-CBCT reconstruction. Nevertheless, residual streaking artifacts, indicated by the blue arrows, were observable around the bony region. In contrast, MA-ROOSTER incorporated additional motion information from the prior 4D-CT.

**Figure 8.**
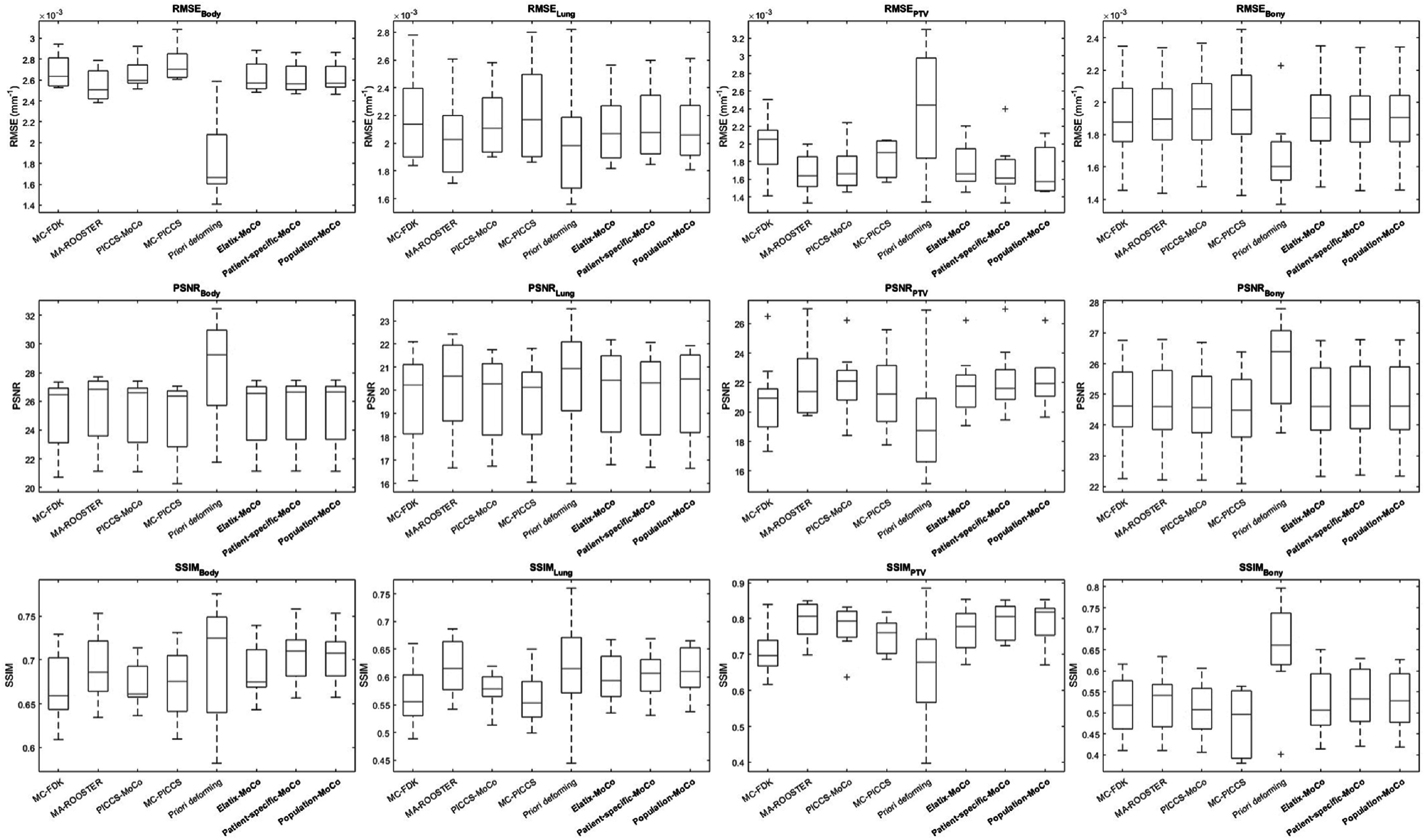
Boxplots of root-mean-square-error (RMSE), peak signal-to-noise ratio (PSNR), and structural similarity index metric (SSIM) values for different reconstruction methods on the SPARE Monte Carlo simulation dataset. The red lines and red plus signs represent the median and outliers, respectively. Our implementations are indicated by bold font.

Quantitative evaluation results of the reconstruction accuracy within four different ROIs for nine Monte Carlo simulation datasets are presented in figure 8. No statistically significant difference was observed among Elastix-MoCo, patient-specific-MoCo, and population-MoCo in terms of RMSE, PSNR, and SSIM in each ROI (one-way ANOVA, ${p}\,{=}{0}{.}{45}{-}{0}{.}{99}$).

The runtime of the entire MoCo reconstruction with motion models estimated using the Elastix DIR (with an Intel Xeon Gold 6226R CPU), patient-specific model, and pre-trained population model is shown in table 2. In our implementations, most of the computation time for Elastix-MoCo was spent during motion modeling, averaging 01:36:26 (hh:mm:ss). In contrast, the average time spent on the motion modeling processes using patient-specific model and population model were 00:09:49 and 00:00:02, respectively.

**Table 2.** Runtime (hh:mm:ss) of the entire 4D-CBCT MoCo reconstruction on simulation dataset with motion models estimated using different methods.

Case	Elastix-MoCo	Patient-specific-MoCo	Population-MoCo
1	01:41:04	00:11:23	00:01:16
2	01:36:55	00:13:13	00:01:01
3	01:42:16	00:12:12	00:01:01
4	01:43:06	00:13:22	00:01:19
5	01:39:24	00:07:32	00:01:02
6	01:23:49	00:10:24	00:01:03
7	01:35:30	00:09:09	00:01:00
8	01:35:02	00:10:32	00:00:59
9	01:39:46	00:11:07	00:01:7
Mean (± std)	01:37:26 (± 00:05:51)	00:10:59 (± 00:01:53)	00:01:06 (± 00:00:08)

### 3.3. Reconstruction results of real patient data

**Figure 9.**
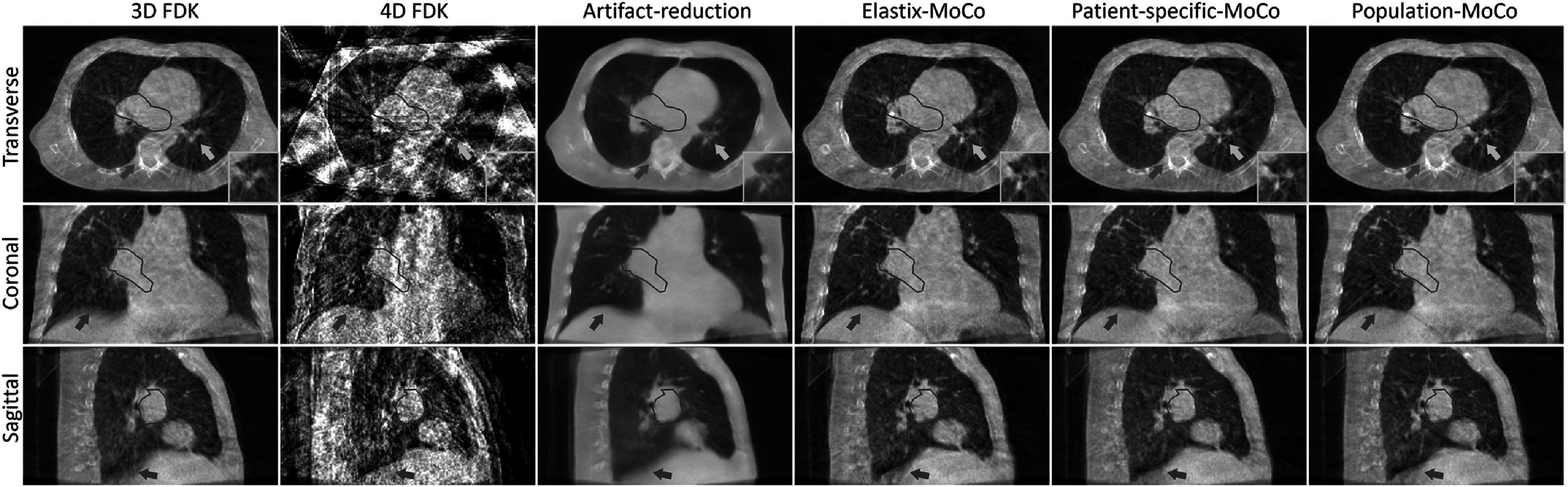
Representative reconstruction results of real patient scan. Tumor regions are indicated by red contours. The display range is [−0.002, 0.028] mm^−1^.

To validate the performance of our proposed method on real patient data, the exemplary reconstruction results of a clinical scan are shown in figure 9. 17 respiratory cycles were contained in the 1-min acquisition. The 3D-CBCT reconstructed using FDK with all available projections is presented for reference in column 1, where the respiratory motion blurring can be observed as indicated by red arrows. The overall observations for this real patient image were similar to those obtained from the simulation dataset. Compared to the artifact-reduced initial image, all MoCo-based reconstructions mitigated the over-smoothness and distortion in the bone structures (blue arrows) and vessels (yellow arrows and zoomed-in images). MoCo reconstructions using DL-based registration exhibited comparable performance to that used traditional registration methods. For this representative case, the runtime (hh:mm:ss) for motion modeling process using the Elastix DIR, patient-specific model, and population model were 01:45:14, 00:09:22 and 00:00:02, respectively. The 4D animations showing the full breathing cycle for this patient are available in the Supplementary Material as Movie S1-S3. The reconstruction results of another patient case are also presented in the Supplementary Material as Movie S4-S6.

## 4. Discussion

In our previous work, we combined DL-based artifact reduction and MoCo to solve the 4D-CBCT reconstruction problem (Zhang *et al* 2023). The results have demonstrated the advantage of CNN-generated artifact-reduced initial images in estimating accurate motion models and improving the subsequent MoCo reconstruction. However, the clinical application can be limited by the slow speed of motion modeling via conventional registration methods. Therefore, in this study, we proposed a MoCo reconstruction framework using DL-based artifact reduction and groupwise registration to simultaneously achieve fast and high-quality 4D-CBCT reconstruction.

The main aim of this study is to develop and investigate DL-based registration methods compatible with near real-time MoCo reconstruction to address the efficiency challenges associated with 4D-CBCT reconstruction. Due to the intrinsic iterative optimization process, the real-time motion modeling via conventional registration methods is impractical. We used a B-spline based groupwise registration implemented in the Elastix toolbox as the reference for conventional methods, given its numerous successful applications. The comparison of registration accuracy and computation time with other advanced conventional registration methods was beyond the scope of this paper. We tested two DL registration models with different learning strategies. The patient-specific model used one-shot learning to train the network parameters only using images to be registered, which eliminated the demand for a large training dataset. The population model was pre-trained on a set of 4D-CT images, allowing direct inference on new images. Table 1 demonstrated that both employed models obtained sufficient registration accuracy on DIR-Lab dataset compared to other state-of the-art methods. The patient-specific model tailored for each individual patient exhibited better registration accuracy than the population model at the expense of increased computation time. Nevertheless, the marginal deviation in registration accuracy did not hamper the final MoCo reconstruction. A potential explanation could be that the artifact-reduced CBCT images used for motion modeling in MoCo exhibited relative smoothness, thereby resulting in a mitigation of the performance disparity between the two models. As shown in figure 8, no significant difference (${p}{> }{0}{.}{05}$) was observed among the use of motion models generated by traditional registration method, patient-specific model, and population model. This demonstrated the feasibility of integrating DL-based registration methods into the MoCo process by replacing conventional registration techniques for motion modeling. The DL-based registration methods achieved sufficient registration accuracy and quality to enable MoCo reconstruction, while prioritizing the rapid computation required for radiotherapy with the patient in the treatment position. Using only the 4D images to be registered as input and employing a one-shot learning strategy, the patient-specific model can generate the motion model more quickly than the conventional DIR method. Furthermore, with access to a sufficient number of high-quality 4D images, the population registration model can be pre-trained, further reducing the duration of motion modeling and the overall MoCo reconstruction process. Additionally, in comparison with five previously proposed methods, although the performance of different methods varied across different ROIs and metrics, our proposed method exhibited noticeable advantages over other methods in the tumor-bearing region. This validated the feasibility of using DL-based groupwise registration for fast MoCo reconstruction. It should also be noted that the population model was trained using 4D-CT images but applied on artifact-reduced 4D-CBCT images for motion modeling. The accurate reconstruction results demonstrated the robustness and generalization of our population model to different modalities. The performance of proposed method was also qualitatively validated on a real patient scan, as shown in figure 9.

Motion modeling is the most time-consuming process in the conventional MoCo reconstruction. The DL-based registration significantly reduced this runtime from hours for the traditional registration method to minutes for patient-specific model and mere seconds for population model. This allowed a nearly real-time MoCo reconstruction for 4D-CBCT. Specifically, there were four main steps in the proposed method, including the use of FDK for initial phase image reconstruction, artifact reduction, image registration and another warped FDK for the finial MoCo reconstruction. Population-MoCo achieved a total reconstruction time of approximately 1 min for SPARE simulation dataset, with all steps but the warped FDK running on GPUs. The future GPU implementation of the warped FDK would further reduce the reconstruction time. The fast reconstruction can not only reduce the treatment duration, but also eliminate the potential errors introduced by patients’ movement during the interval between 4D-CBCT acquisition and MoCo image availability.

There have been other works following a similar methodology to our previous work, leveraging DL-based artifact reduction to enhance the MoCo reconstruction for 4D-CBCT. For example, Yang *et al* (2023) used a multiscale-discriminator generative adversarial network to alleviate streaking artifacts in initial images. Ou *et al* (2024) incorporated prior knowledge derived from all projections into the network to enhance the structure restoration while reducing streaking artifacts. The approach we evaluated here could be integrated with these methods by using DL-based DIR to estimate the motion model and accelerate MoCo reconstruction. In addition, our proposed workflow does not impose any requirements on the architecture or type of the DIR model. Other registration models could also be plugged into the workflow to achieve fast MoCo reconstruction. However, exploring the optimal DIR model is out of our current consideration.

The current study implemented MoCo reconstruction for 4D-CBCT using analytical reconstruction (i.e. FDK) and two separately trained networks dedicated to artifact reduction and DIR. There might be potential advantages in merging all components into a trainable end-to-end loop. However, a major challenge for this strategy is the limitation of GPU memory. End-to-end training requires the integration of the projection data and system matrix into the CNN, which can be almost impossible for a conventional CBCT scan due to the huge size. Additionally, a large number of ground truth 4D-CBCT images will be necessary for the end-to-end training. This requirement can also be challenging to fulfill in clinical setting. Such attempts will be deferred to the future.

## 5. Conclusion

In this study, we proposed to use DL-based groupwise registrations to speed up the motion modeling for MoCo reconstruction based on artifact-reduced initial images. The feasibility was validated using both Monte Carlo simulation data from the publicly available SPARE challenge and real patient data. The proposed method using the pre-trained population registration model for motion modeling achieved a total reconstruction time of approximately 1 min without compromising image quality, which facilitates the clinical deployment of 4D-CBCT for image-guided radiation therapy.

## Data Availability

The data cannot be made publicly available upon publication because they contain sensitive personal information. The data that support the findings of this study are available upon reasonable request from the authors.
